# Small molecules block the interaction between porcine reproductive and respiratory syndrome virus and CD163 receptor and the infection of pig cells

**DOI:** 10.1186/s12985-020-01361-7

**Published:** 2020-07-30

**Authors:** Chang Huang, Denzil Bernard, Jiaqi Zhu, Radha Charan Dash, Alexander Chu, Alec Knupp, Anna Hakey, M. Kyle Hadden, Antonio Garmendia, Young Tang

**Affiliations:** 1 Department of Animal Science, Institute for Systems Genomics, University of Connecticut, 1390 Storrs Rd, Storrs, CT 06269 USA; 2 Atomwise Inc, 717 Market Street, Suite 800, San Francisco, CA 94103 USA; 3 Department of Pharmaceutical Sciences, University of Connecticut, 69 North Eagleville Rd, Storrs, CT 06029 USA; 4 Department of Pathobiology and Veterinary Sciences, University of Connecticut, 61 North Eagleville Road, Storrs, CT 06269 USA

**Keywords:** Porcine reproductive and respiratory syndrome (PRRS), PRRS viruses (PRRSV), CD163, Protein-protein interaction (PPI), Scavenger receptor cysteine-rich domain 5 (SRCR5), Bimolecular fluorescence complementation (BiFC), Porcine alveolar macrophages (PAMs)

## Abstract

**Background:**

Porcine reproductive and respiratory syndrome (PRRS) is one of the most economically devastating diseases affecting the pork industry globally. PRRS is caused by PRRS virus (PRRSV). Currently there are no effective treatments against this swine disease.

**Methods:**

Through artificial intelligence molecular screening, we obtained a set of small molecule compounds predicted to target the scavenger receptor cysteine-rich domain 5 (SRCR5) of CD163, which is a cell surface receptor specific for PRRSV infection. These compounds were screened using a cell-based bimolecular fluorescence complementation (BiFC) assay, and the function of positive hit was further evaluated and validated by PRRSV-infection assay using porcine alveolar macrophages (PAMs).

**Results:**

Using the BiFC assay, we identified one compound with previously unverified function, 4-Fluoro-2-methyl-N-[3-(3-morpholin-4-ylsulfonylanilino)quinoxalin-2-yl]benzenesulfonamide (designated here as B7), that significantly inhibits the interaction between the PRRSV glycoprotein (GP2a or GP4) and the CD163-SRCR5 domain. We further demonstrated that compound B7 inhibits PRRSV infection of PAMs, the primary target of PRRSV in a dose-dependent manner. B7 significantly inhibited the infection caused by both type I and type II PRRSV strains. Further comparison and functional evaluation of chemical compounds structurally related to B7 revealed that the 3-(morpholinosulfonyl)aniline moiety of B7 or the 3-(piperidinylsulfonyl)aniline moiety in a B7 analogue is important for the inhibitory function against PRRSV infection.

**Conclusions:**

Our study identified a novel strategy to potentially prevent PRRSV infection in pigs by blocking the PRRSV-CD163 interaction with small molecules.

## Introduction

Porcine reproductive and respiratory syndrome (PRRS) is one of the most economically significant swine diseases, with over billion-dollar losses to the global pork industry annually. The causative virus of PRRS (PRRSV) is an enveloped, positive-sense, single-stranded RNA virus of the *Arterivirus* genus within the order *Nidovirales* [1, 2]. PRRSV infection results in severe reproductive failure in sows and respiratory disease in piglets [3]. This may be complicated by secondary infections with even greater clinical manifestations and mortality [4–6]. Unfortunately, due to the high genetic and antigenic heterogeneity of PRRSV, broadly effective vaccines are still lacking [7–9]. New approaches are needed to combat the PRRS panzootic to mitigate the devastating consequences of this disease.

The productive PRRSV infection occurs primarily through porcine alveolar macrophages (PAMs) in the pig lung [10]. CD163, a macrophage-specific membrane scavenger receptor, is a key receptor for PRRSV infection [11–14]. The necessity of CD163 expression for PRRSV infection was confirmed by knockout studies showing pigs without CD163 become PRRSV-resistant [15–17]. Out of the 9 extracellular scavenger receptor cysteine-rich (SRCR) domains in CD163, SRCR5 was found crucial for PRRSV infection [18], and monocytes/macrophages from pigs expressing CD163 with deleted SRCR5 are fully protected from PRRSV infection [19]. Cellular pull-down assay and bimolecular fluorescence complementation (BiFC) analysis revealed that PRRSV directly interacts with CD163 via its minor glycoproteins GP2a and GP4 [20, 21], which bind the CD163 extracellular but not transmembrane or cytoplasmic region [21]. Thus, it is reasonable to assume that the CD163-SRCR5 domain directly interacts with the PRRSV glycoproteins. However, assays studying protein-protein interactions (PPIs) between the CD163-SRCR5 domain and PRRSV glycoproteins have not been reported.

A number of small molecules have been identified to effectively block the entry of various human viruses by binding and antagonizing the host cell receptors/co-receptors [22–29]. However, a small molecule targeting the PPI between PRRSV and CD163 has not been reported. A recent study of the porcine CD163 X-ray crystal structure revealed a distinct 3-D structural arrangement of the CD163-SRCR5 domain loop 5–6 region (Phe544-Arg570) compared to its homologous region in SRCR-superfamily proteins M2BP and CD5 [30]. Furthermore, a CD163 mutant with Arg561 changed to Ala in the loop 5–6 region of SRCR5 inhibited PRRSV infection compared with the wild type CD163 [30]. This raises the possibility that targeting pig CD163-SRCR5 at the Arg561 region with small molecules may prevent PRRSV infection. In this study, we developed a BiFC assay to study the PPI between PRRSV glycoproteins and the CD163-SRCR5 domain. Using this assay, we were able to screen a list of small molecules predicted to bind the pig CD163-SRCR5 domain by AtomNet [31] to identify compounds that inhibit the PPI between PRRSV glycoproteins and SRCR5. We further validated the ability of the positive compound to inhibit PRRSV infection of PAMs in vitro. Analyzing B7 and a few of its analogues revealed functional moieties important for the inhibitory activity of B7 against PRRSV infection.

## Materials and methods

### Chemicals, cells, and viruses

All screening compounds were provided by Atomwise, Inc. (CA, USA) as part of the Artificial Intelligence Molecular Screen (AIMS) awards program through Mcule, Inc. (CA, USA), or purchased directly from MolPort, Inc. (NY, USA). PAMs were harvested from 6 healthy 4–6-month old and PRRSV-negative Landrace/Yorkshire cross pigs. Briefly, pigs were euthanized before slaughtered. Lungs were transferred on ice to a cell culture cabinet. Carefully injected warm PBS with 200 U/mL penicillin and 200 μg/mL streptomycin through trachea bronchi into both sides of the lungs. Massaged and then retrieved bronchoalveolar lavage fluid (BALF). The BALF was centrifuged at 400 g for 15 min, the pellets were PAMs. The pellets were then washed twice with warm complete medium. Cells were counted and frozen in 90% FBS (HI, Rocky Mountain Biologicals, Inc) and 10% DMSO (Sigma). Cells were stored in Mr. Frosty Freezing Container (Nalgene, USA), and put at − 80 °C overnight before transferred to liquid nitrogen. PAMs were cultivated in RPMI-1640 (Gibco) supplemented with 10% FBS (HI, Rocky Mountain Biologicals, Inc), 2 mM Glutamax (Invitrogen), 0.1 mM MEM Non-Essential Amino Acids (Gibco), 1 mM sodium pyruvate (Gibco), 100 U/mL penicillin and 100 μg/mL streptomycin (Gibco), and 0.5 μg/mL Amphotericin B (Gibco). All PRRSV strains were propagated and titrated in MARC-145 cells.

### AIMS screen

Virtual screening was performed using AtomNet, the first deep neural network for structure-based drug design trained to predict protein-ligand binding affinity [31]. For targeting the interaction between the porcine CD163 and PRRSV glycoprotein (GP2a or GP4), the X-ray structure of CD163-SRCR5 domain (PDBID:5HRJ) was used to define a screening site centered around R561 comprising residues C502, S503, D505, W540, A541, E543, A559, P560, R561, P562, D563, G564, and C566. The Mcule library of commercially available organic small molecule compounds (~ 4 M v20171018) was prepared and screened, as described previously [32], using an ensemble of protein-ligand conformations. Each of the 4 M molecules was scored and ranked by AtomNet, following which a top set of 200 chemically diverse compounds was further inspected for undesirable substructures and molecular properties before 74 compounds were obtained for experimental testing.

### Plasmid construction

N terminus and C terminus of the truncated Venus-I152L were inserted into vector backbone pMyc-CMV and pCMV-HA, constituting commercial plasmids pBiFC-VN155(I152L) and pBiFC-VC155, respectively (Addgene, Watertown, MA, USA) (Kodama, Y. et al., Biotechniques, 2010). cDNA fragments for the scavenger receptor cysteine-rich domain 2 (SRCR2) and SRCR5 of porcine CD163 receptor, and for glycoproteins GP2a and GP4 of PRRSV VR-2332 strain were amplified by reverse transcription-polymerase chain reaction (RT-PCR). The amplified cDNA fragments of SRCR2 or SRCR5 were subcloned into the pBiFC-VN155(I152L) vector digested with EcoRI/BglII. For constructions of GP2a and GP4 fusion proteins, cDNA fragments encoding GP2a or GP4 were subcloned into the pBiFC-VC155 vector digested with EcoRI/BglII. All plasmid clones were verified by DNA Sanger sequencing.

### BiFC assay

HEK293T cells cultured in 12-well plates were transfected with appropriate plasmids for each BiFC assay using FuGENE® 6 (Promega, Madison, WI, USA). After 5 h, various concentrations of chemical compounds were added to the culture media. DMSO was used as the vehicle control. Fluorescence images of treated cells at 24 h after plasmid transfection were captured using an inverted Nikon fluorescence microscope. Fluorescence intensity of treated cells was measured by ImageJ (https://imagej.nih.gov/ij/).

### Cytotoxicity assay

The cytotoxicity of selected screening compounds was determined in PAMs, the principal host cell of productive PRRSV infection. Briefly, various concentrations of the compounds were added to PAMs seeded in 24-well plates and incubated for 24 h. Then 50 μl of the MTT assay labeling reagent (In Vitro Toxicology Assay Kit, MTT based, Sigma-Aldrich) were added to each well and incubated for 4 h before 500 μl of the solubilization solution was added into each well to fully dissolve the formazan crystal by overnight incubation. The absorbance of samples was measured using a spectrophotometry microplate reader at 600 nm. PAMs treated with DMSO served as the controls.

### Quantitative reverse transcription – PCR (qRT-PCR)

Total RNA was extracted from PAMs infected with PRRSV using RNeasy Mini Kit (Qiagen, Germantown, MD) according to the manufacturer’s instruction. RNA concentrations were measured using a Nanodrop spectrophotometer (Thermo Fisher Scientific). Specific qRT-PCR primers for the ORF7 gene of the four PRRSV strains and for porcine GAPDH are shown in Table S2. GAPDH was used as the housekeeping gene for gene expression normalization. Data were processed with the software associated with ABI 7500.

### Western blotting

Whole cell proteins were isolated from HEK293T cells. Briefly, the cells were rinsed twice with cold PBS. After removing the PBS, cold RIPA buffer (ThermoFisher Scientific) supplemented with 1% (v/v) protease and phosphatase inhibitors was added to the cells and placed on ice for 5 min. Protein concentrations were determined using Pierce BCA Protein Assay Kit (ThermoFisher Scientific). Equal amount of denatured proteins from each sample were separated on 10% SDS-PAGE and transferred onto a PVDF membrane. The membrane was incubated with 5% skim milk to block nonspecific binding before incubation with Myc-Tag mouse monoclonal antibody (1:1000, Cell Signaling Technology, Danvers, MA) or anti-GAPDH antibody (1:1000; Cell Signaling Technology, Danvers, MA) overnight at 4 °C. After washing with 1× T-BST, the membrane was incubated with HRP-conjugated secondary antibody (Cell Signaling Technology, Danvers, MA) for 1 h at room temperature, and images developed with ECL Blotting Substrates (Bio-Rad) were visualized under the ChemiDox XRS Image System (Bio-Rad).

### PRRSV infection and titration assay

For PRRSV infection of PAM cells, PAMs were seeded one day prior to infection. Control cells were treated with DMSO and test cells were treated with the selected screening compounds (5–20 μM) for 4 h before inoculation, during the 1 h PRRSV inoculation, and for 24 h after inoculation. Cells were inoculated with VR-2332, SDSU73, NADC30 or Lelystad PRRSV at MOI = 0.1 for 1 h. The 24 h cell medium supernatant was stored at − 80 °C until future titration assay.

For PRRSV titration assay, MARC145 cells were seeded in 48-well plates and grew to ~ 80% density before inoculation. Viral supernatants were prepared by 10-fold serial dilution, and 100 μl of the dilutions was added per well in six replicates. Inoculum was removed from cells after 2 h and replaced with 0.5 mL of DMEM supplemented with 2% FBS, 2 mM Glutamine, 0.1 mM MEM Non-Essential Amino Acids, and 50 U/mL penicillin and 50 μg/mL streptomycin (Invitrogen) to each well. Cells were cultured at 37 °C for 6 days and then the cytopathic effects were recorded. The PRRSV titer was calculated using the Reid and Müench method and expressed as median tissue culture infectious dose (TCID_50_/mL).

### Statistical analysis

All experiments were performed at least 3 times. Data were analyzed by one-way ANOVA with Tukey’s post hoc comparison or by paired t-test. Data were expressed as mean ± sd and *p* < 0.05 was considered to be significant.

## Results

### Development of BiFC assays to identify small molecules that inhibit the PPI between PRRSV and CD163

**Fig. 1:**
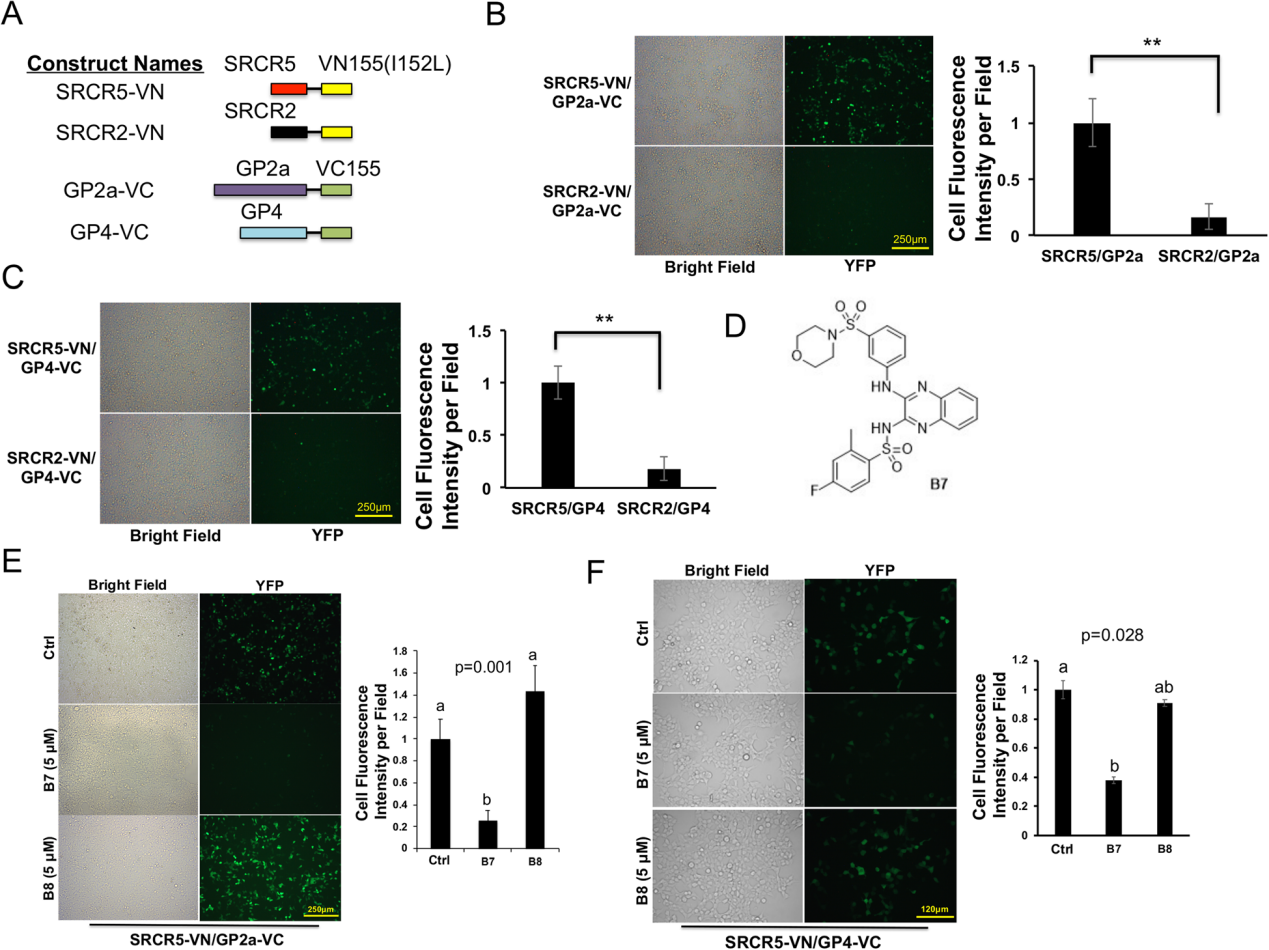
Establishing BiFC Assays and Screening Compounds that Inhibit the PPI Between PRRSV and CD163. **a** Scheme diagram for the BiFC assay fusion protein constructs between Pig CD163 SRCRs or PRRSV minor envelope glycoproteins and the fragments of Venus protein VN155(I152L) or VC155), respectively. **b** Left: SRCR5-VN or SRCR2-VN plasmid was co-transfected with GP2a-VC to HEK293T cells, with fluorescent images taken at 24 h after transfection. Bar = 250 μm. Right: Cell fluorescence quantified by Fiji. Mean ± SD. *n* = 3. **: *p* < 0.01. **c** Left: SRCR5-VN or SRCR2-VN plasmid was co-transfected with GP4-VC to HEK293T cells, with fluorescent images taken at 24 h after transfection. Bar = 250 μm. Right: Relative fluorescence intensity quantified. Mean ± SD. *n* = 3. **: p < 0.01. **d** Chemical structure of compound B7. **e** Left: BiFC assay between SRCR5-VN and GP2a-VC proteins. Images showing positive inhibitory effect of compound B7 but not B8, with DMSO as the Ctrl. Bar = 250 μm; Right: Relative fluorescence intensity quantified. Mean ± SD, n = 3. **f** Left: BiFC assay between SRCR5-VN and GP4-VC proteins showing similar inhibitory effect by B7 compound but not by B8. Bar = 120 μm. Right: Relative fluorescence intensity quantified. Mean ± SD, n = 3

Using the previously described BiFC vector [33] based on the fragmented Venus protein (VN-155[I152L], hereafter named VN) and (VC-155, hereafter named VC), we established fusion protein constructs between the porcine CD163 protein SRCR5 or SRCR2 domain and VN, and between the PRRSV minor glycoproteins (GP2a or GP4) and VC (Figs. 1a, S1). We co-expressed these plasmids in HEK293T cells to evaluate the PPIs between the SRCR5 domain and GP2a or GP4. In agreement with the demonstrated critical role of CD163-SRCR5 in mediating the PRRSV-CD163 interaction and thus PRRSV infection [18, 19], the CD163-SRCR5/VN fusion protein (SRCR5-VN) interacts with GP2a- or GP4-VC, with strong fluorescence detected under the microscope (Fig. 1B, C, top panel). In contrast, the fusion protein of CD163-SRCR2 domain (SRCR2-VN), which is dispensable for the PRRSV infection and PRRSV-CD163 interaction [18], only showed background fluorescence when co-expressed with GP2a- or GP4-VC (Fig. 1b, c, lower panel). These data support that the porcine CD163-SRCR5 domain interacts directly with PRRSV glycoproteins GP2a and GP4.

We then asked whether the BiFC assays we established could be used to identify small molecules with potential to block the interaction between CD163 and PRRSV. A customized virtual screening of a library of commercially available small molecules was performed with AtomNet for a region centered around R561 (Fig. S2) based on the available 3-D protein structure of porcine CD163-SRCR5 domain [30], and 74 compounds predicted to target this potential binding region were procured. We then tested these compounds using our BiFC assay, to evaluate their ability to inhibit the PPI between GP2a and SRCR5. Plasmids were co-transfected in HEK293T cells, 4 h later, chemicals were applied individually to cells at 5 μM. Twenty-four h later, images were taken under a fluorescence microscope and the fluorescence was quantified. Of all 74 compounds, we identified 1 positive hit named herein as B7 (4-Fluoro-2-methyl-N-[3-(3-morpholin-4-ylsulfonylanilino)quinoxalin-2-yl]benzenesulfonamide, C_25_H_24_FN_5_O_5_S_2_, Fig. 1d, Table S1) that significantly inhibited the reconstituted fluorescence in our BiFC assay (Fig. 1e). Using the other BiFC assay, we further verified that B7 also inhibited the PPI between PRRSV GP4 glycoprotein and CD163-SRCR5 domain (Fig. 1f).

### Inhibition of PRRSV infection of PAMs by compound B7

**Fig. 2:**
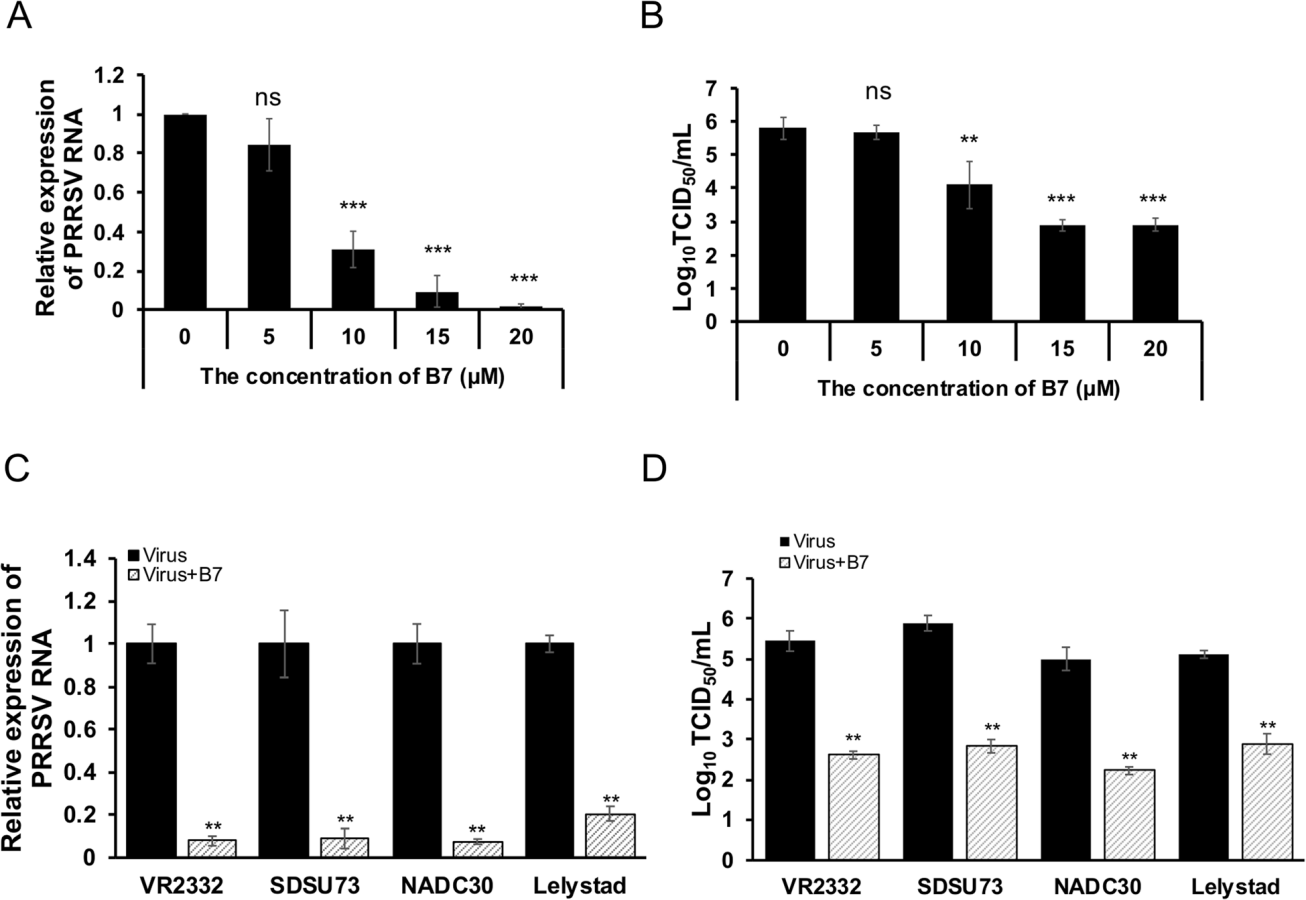
Inhibition of the PRRSV Infection of PAMs by Compound B7. **a** qRT-PCR for PRRSV in total RNAs extracted from PRRSV strain VR-2332 infected PAMs treated with various concentrations of B7 compound. Values are normalized with GAPDH of PAMs. Bars = mean ± SD, n = 3. ***: *P* < 0.001. ns: non-significant. **b** Titration assay results for PRRSV in the culture media of PAMs treated as described in (**a**). Bars = mean ± SD, n = 3. **: *P* < 0.01, ***: P < 0.001. ns: non-significant. **c** qRT-PCR for PRRSV in total RNAs extracted from PAMs infected by different strains of PRRSV and treated with 15 μM B7 compound. Values are normalized with GAPDH of PAMs. Bars = mean ± SD, n = 3. **: *P* < 0.01. **d** Titration assay results for PRRSV in the culture media of PAMs treated as described in (**c**). Bars = mean ± SD, n = 3. **: P < 0.01

Based on the demonstrated inhibitory effect of B7 on the interaction between CD163-SRCR5 domain and PRRSV glycoproteins, we asked whether this compound will inhibit the PRRSV infection of PAMs. MTT assay revealed that the B7 compound is well-tolerated by PAMs at concentrations below 25 μM, with the LC_50_ calculated [34] to be 81.7 μM (Fig. S3). Primary PAMs were then pre-treated with B7 at 0, 5, 10, 15, or 20 μM for 4 h, followed by 1 h inoculation with PRRSV strain VR-2332 (MOI = 0.1). The infected PAMs were then continuously incubated with different concentrations of B7. At 24 h after inoculation, total RNAs were extracted from PAMs. Quantitative reverse transcription – PCR (qRT-PCR) revealed a dose-dependent inhibition of PRRSV infection of PAMs by the B7 treatment (Fig. 2a). Titration of PRRSV from the culture media of the infected PAMs further confirmed that 10–20 μM B7 inhibited PRRSV infection of PAMs effectively, with 1.7 and 2.9 log reduction of viral titer by 10 and 15 μM B7 treatment, respectively (Fig. 2b), with the calculated IC50 value of 7.5 μM for B7. The selective index is a value that measures the window between drug cytotoxicity and selective activity, which was estimated to be 10.9 for B7 compound from this study based on the ratio of LC_50_/IC_50_ similarly as described previously [35].

The inhibitory effect of B7 was further tested on multiple strains of PRRSV. PAMs were pre-treated with 15 μM B7 for 4 h, and inoculated with PRRSV strains VR-2332, SDSU73, NADC30 (Type II), or Lelystad (Type I) for 1 h, and continuously incubated with 15 μM B7 for 24 h. qRT-PCR revealed that B7 treatment significantly decreased the viral RNA detected in infected PAMs by all PRRSV strains (Fig. 2c). This inhibitory effect is further confirmed by viral titration results for the culture media of the infected PAMs, with 2.8–3.1 log reduction of viral titers across the 3 Type II PRRSV strains and 2.2 log reduction of viral titer for the type I strain (Lelystad) by 15 μM B7 treatment (Fig. 2d). Thus, compound B7 indeed inhibits the PRRSV infection of PAMs.

The AtomNet platform screening based on protein-ligand conformation and our BiFC assay results indicated a direct binding of B7 with CD163-SRCR5 rather than binding individually with GP2a and GP4 to block the CD163-PRRSV interaction. To further exclude the possibility that B7 targets PRRSV glycoproteins directly, we incubated stock virus of PRRSV VR-2332 strain with 15 μM B7 or DMSO followed by a direct viral titration assay. If B7 directly targets PRRSV glycoproteins, the virus pre-incubated with B7 should have decreased infectivity compared with the control. However, no significant reduction in viral titer was observed (Fig. S4). Taken together, our results support that compound B7 inhibits PRRSV infection by targeting CD163-SRCR5 domain.

### Evaluating the PRRSV infection inhibitory effect for compounds with similar chemical structure as B7

**Fig. 3:**
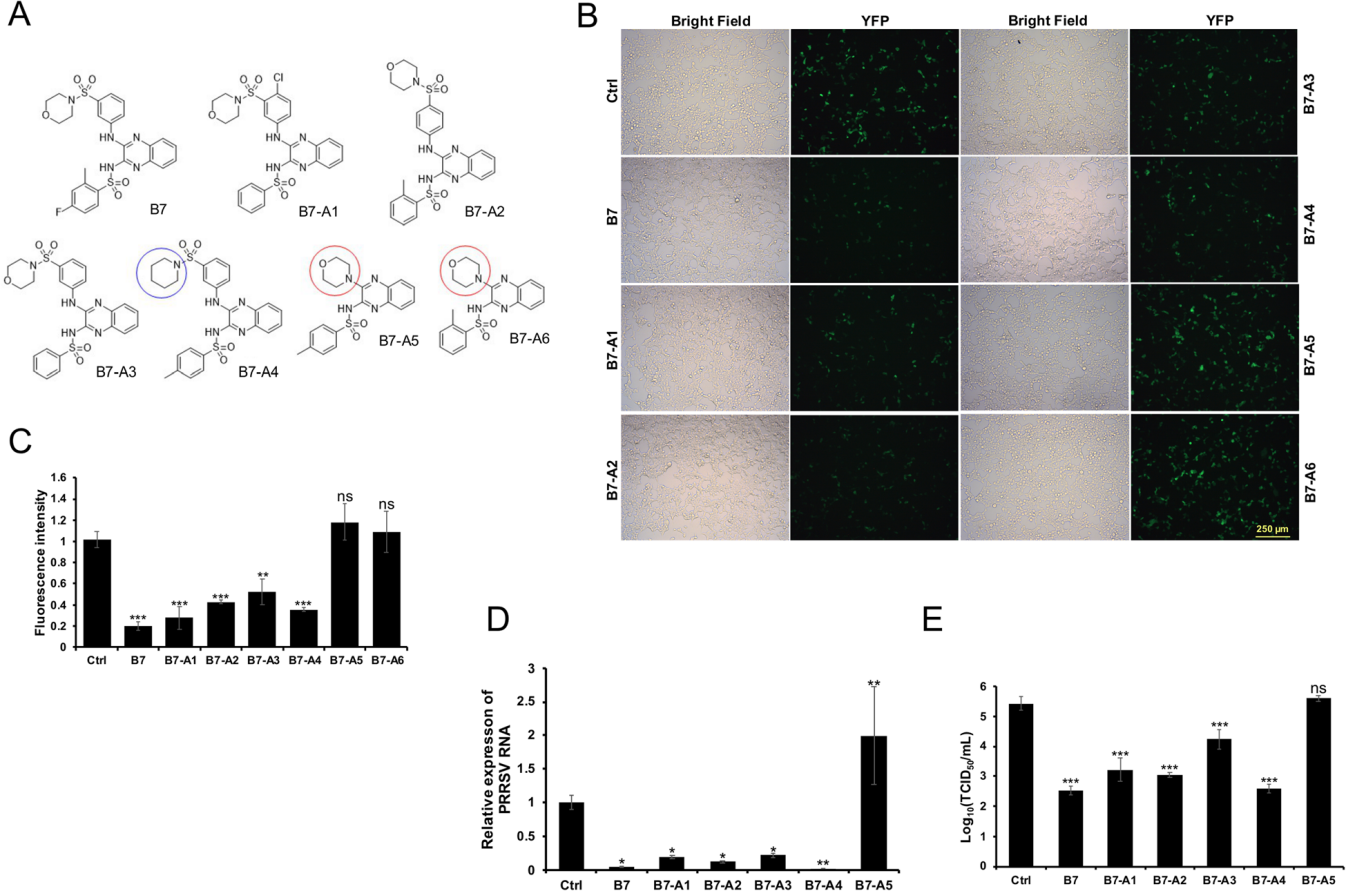
Evaluating the Inhibitory Effect on PRRSV Infection by Compounds Structurally Similar to B7. **a** Molecular structures of B7 analogues (B7-A1 to A6) compared with B7. **b** BiFC assay between SRCR5-VN and GP2a-VC proteins. Representative fluorescent images showing different effects between compound B7 and its analogues on the SRCR5-GP2a PPI, with DMSO as the Ctrl. Bar = 250 μm. **c** Relative fluorescence intensity in (**b**) quantified by Fiji. Mean ± SD, n = 3. **: P < 0.01, ***: P < 0.001. ns: non-significant. **d** qRT-PCR for PRRSV in total RNAs extracted from PRRSV strain VR-2332 infected PAMs treated with 15 μM of B7 and its analogues. Values are normalized with GAPDH of PAMs. Bars = mean ± SD, n = 3. *: *P* < 0.05, **: P < 0.01. **e **Titration assay results for PRRSV in the culture media of PAMs treated as described in (**d**). Bars = mean ± SD, n = 3. *: P < 0.05, **: P < 0.01. ns: non-significant

B7 is a synthetic compound with a previously unverified function. In order to examine the structural relevance of the B7 scaffold to PRRSV infection inhibition, we performed PubChem search for similar compounds and obtained an additional 6 compounds structurally related to B7 (named herein as B7-A1 to A6, Fig. 3a, Table S1). We first used our BiFC assay to check if any of these B7 analogues can inhibit the PPI between CD163-SRCR5 domain and PRRSV GP2a protein. Similar to B7, compounds B7-A1 to B7-A4 all significantly inhibited the CD163-SRCR5/GP2a PPI in our BiFC assay (Fig. 3b, c). Interestingly, shifting the 3-(morpholinosulfonyl)anilino moiety of B7 to 4-(morpholinosulfonyl)anilino position (B7-A2), or changing the former moiety to 3-(piperidinylsulfonyl)anilino group (B7-A4, blue circle) does not eliminate these compounds’ ability to inhibit the CD163-SRCR5/GP2a PPI (Fig. 3b, c). However, replacing the 3-(piperidinylsulfonyl)anilino or 3-(morpholinosulfonyl)anilino moiety by morpholine in compounds B7-A5 and B7-A6 (Fig. 3a, red circle) completely blocked their ability to inhibit the CD163-SRCR5/GP2a interaction (Fig. 3b, c).

In order to confirm the function of these compounds, we further evaluated compounds B7-A1 through B7-A5 on PRRSV infection of PAMs. MTT assay revealed that these compounds have no obvious cytotoxic effect on PAMs at 15 μM level (Fig. S5). PAMs were then pre-treated with 15 μM of each compound for 4 h and inoculated with PRRSV strain VR-2332 for 1 h. The cells were then continuously incubated with 15 μM of the same compound. At 24 h, total RNAs were extracted from the infected PAMs and qRT-PCR demonstrated that similar to B7, compounds B7-A1 to B7-A4, all significantly inhibited the PRRSV RNA level in the treated PAMs compared with the control (Fig. 3d). Also, consistent with our BiFC results, treatment with B7-A5 failed to inhibit PRRSV RNA level in the infected PAMs (Fig. 3d). Titration of the 24 h culture media of infected cells further confirmed our finding by demonstrating that compounds B7-A1 through B7-A4 but not B7-A5 all inhibited PRRSV infection of PAMs, with 2.2, 2.4, 1.2, and 2.8 log reduction of viral titer by compounds B7-A1 through A4 treatment, respectively (Fig. 3e). Taken together, our results indicate that the 3-(morpholinosulfonyl)anilino moiety (in B7 and B7-A1 through B7-A3) and the 3-(piperidinylsulfonyl)anilino moiety (in B7-A4) are important for the inhibitory effect of these compounds on PRRSV infection.

### 3-(morpholinosulfonyl)anilino or 3-(piperidinylsulfonyl)anilino alone does not inhibit PRRSV infection and post-treatment with B7 significantly inhibits PRRSV infection

**Fig. 4:**
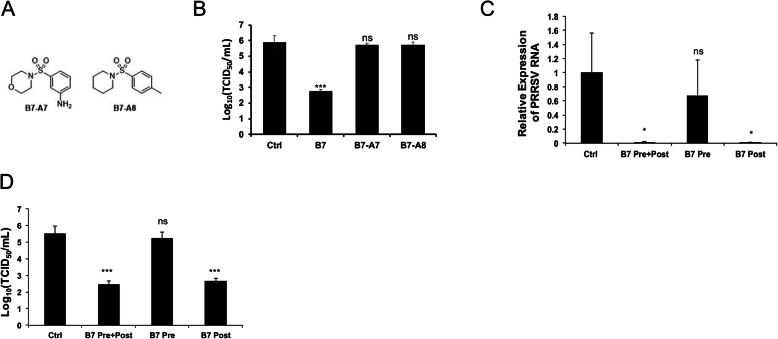
3-(morpholinosulfonyl)anilino or 3-(piperidinylsulfonyl)anilino Alone Does Not Inhibit PRRSV Infection and B7 Post-Treatment Significantly Inhibits PRRSV Infection. **a** Molecular structures of B7-A7 and B7-A8. **b** Titration assay results for the culture media of PAMs treated with B7, B7-A7, or B7-A8 and infected by PRRSV strain V-2332. Bars = mean ± SD, n = 3. ***: P < 0.001. ns: non-significant. **c** qRT-PCR for PRRSV in total RNAs extracted from PAMs infected by PRRSV VR-2332 and treated with 15 μM of B7 compound at pre and/or post-inoculation. Values are normalized with GAPDH of PAMs. Bars = mean ± SD, n = 3. *: *P* < 0.05. ns: non-significant. **d** Titration assay results for PRRSV in the culture media of PAMs treated as described in (**c**). Bars = mean ± SD, n = 3. ***: P < 0.001. ns: non-significant

We also noticed that removing the methyl and/or fluoro groups from the benzenesulfonamide moiety of B7 (compound B7-A1, A2, and A3, Fig. 3a) weakened the compound’s inhibitory function in BiFC assay (Fig. 3b, c) and PRRSV infection assay (Fig. 3d, e), although the effect is not as dramatic as modifying the 3-(morpholinosulfonyl)anilino moiety. In order to determine if the 3-(morpholinosulfonyl)anilino or 3-(piperidinylsulfonyl)anilino chemical group alone would be sufficient to suppress PRRSV infection, we purchased these 2 compounds and named them B7-A7 and B7-A8, respectively (Fig. 4a). We treated PAMs with 15 μM of each molecule for 4 h then followed by 1 h PRRSV VR-2332 inoculation. The cells were then incubated with the same compound for 24 h. Titration of PRRSV from the culture media of treated cells revealed that neither of these 2 compounds inhibited PRRSV infection of PAMs (Fig. 4b). Therefore, while these 2 moieties are functionally critical, the presence of other moieties (e.g., benzenesulfonamide) are also essential for the inhibition of PRRSV infection.

Having verified the inhibitory effect of B7 on PRRSV infection upon pre- plus post-inoculation treatment, we further asked whether treatment with B7 either pre- or post-inoculation alone would have a significant effect. PAMs were treated either with B7 for 4 h only and followed by 1 h PRRSV strain VR-2332 inoculation with no post-treatment, or with only 24 h post-PRRSV inoculation treatment without pre-treatment. At 24 h post infection, we evaluated viral RNA in PAMs and the PRRSV titer in culture media. qRT-PCR revealed that while post-treatment alone by B7 exhibited similar inhibitory effect as the pre- plus post-treatment on PRRSV infection, while pre-treatment had minimum impact (Fig. 4c). This is further confirmed by the viral titration for the culture media of infected PAMs, with compound B7 post-inoculation treatment alone resulted in 2.8 log reduction of PRRSV titer (Fig. 4d).

Previous studies revealed that CD163 mediates PRRSV uncoating instead of internalization during productive infection [36–38], which is a step after virion internalization to the host cells. PRRSV binding and internalization, however, are mediated by other receptor/co-receptors, e.g. heparan sulphate and Sialoadhesin [39]. We performed additional experiment to verify if B7 affects PRRSV binding, by incubating PAMs with 15 μM B7 or DMSO control during the 1 h PRRSV inoculation period. The PRRSV binding of PAMs was evaluated with qRT-PCR immediately after inoculation and repeated wash to remove unbound viruses. We found that B7 incubation during the 1 h inoculation period does not affect PRRSV binding (Fig. S6). Also, we already discovered that B7 post-inoculation treatment exerts comparable anti-PRRSV effect as the pre- plus post-inoculation treatment (Fig. 4c, d). Taken together, our results here are consistent with the mechanism discovered previously that CD163 interacts with PRRSV GP2a and GP4 to mediate PRRSV uncoating after endosome acidification to ensure effective viral infection, rather than mediating the viral binding and internalization [39].

## Discussion

Although the porcine CD163-SRCR5 domain was identified to be critical for PRRSV infection, suggesting a direct interaction between PRRSV and the SRCR5 domain, no study has been reported to verify this particular PPI. Using BiFC assay, we demonstrated that the CD163-SRCR5 domain can interact directly with PRRSV glycoproteins GP2a and GP4. This correlates with the recent reports that genetically modified pigs lacking CD163-SRCR5 domain are resistant to PRRSV infection [40, 41]. We further identified that a small molecule B7, which can block the interaction between CD163-SRCR5 domain and PRRSV glycoproteins, also inhibits the PRRSV infection of PAMs in vitro. The BiFC assays we reported here would be of great value for further screening of small molecules that could block the PPIs between PRRSV and CD163.

We noticed that 5 μM B7 is sufficient to inhibit the interaction between GP2a/GP4a and CD163-SRCR5 (Fig. 1e, f), whereas higher concentrations are needed for significant inhibition of viral replication (Fig. 2a, b). We reasoned that the difference could stem from the fact that B7 compound worked under very different scenarios in BiFC and PRRSV infection assays, respectively. First, in the BiFC assay, only a single PRRSV glycoprotein, GP2a or GP4 was expressed as a fusion protein. However, in the PRRSV infection assay, both GP2a and GP4 were inherently expressed on virion envelop. Second, PAMs were used in PRRSV infection assay, which are very different from the transformed HEK293T cell lines used in BiFC assays. All these could contribute to the assay difference in sensitivity to B7 compound.

The interactions among the 3 PRRSV minor envelope glycoproteins GP2a, GP3, and GP4 are needed for PRRSV infectivity [21, 42]. Also, GP5 is the indispensable major glycoprotein in PRRSV envelope essential for the viral particle production [42]. Although all PRRSV glycoproteins show certain interaction with each other, only GP2a and GP4 specifically interact with the CD163 receptor [21]. Therefore, our study mainly focused on the investigation of blocking the interaction between CD163 and GP2a/GP4 here. Nevertheless, GP5 constitutes an indispensable part of the viral envelope, which may be critical for PRRSV replication in infected cells [20]. Recently it was also shown that GP5 interacts with non-muscle myosin-heavy chain 9, which is important for PRRSV infection through viral internalization mechanism [43]. Previous studies have revealed that CD163 mediates PRRSV uncoating instead of internalization for productive viral infection [36–38]. Thus, it is reasonable for us to speculate here that GP2a/4 and GP5 mediate different and subsequent processes during the viral infection.

We found that the B7 post-inoculation treatment alone yielded a similar degree of inhibition on PRRSV infection compared with the pre- plus post-inoculation treatment, while the pre-inoculation treatment alone had no obvious effect. This indicates a reversible association between B7 compound and the CD163-SRCR5 protein domain, and that the inhibitory effect on  the interaction between PRRSV glycoproteins and CD163-SRCR5 could be lifted upon the removal of B7 compound by washing. It would be interesting to perform further refined structure activity relationship (SAR) analysis to unveil the nature of B7 and SRCR5 molecular-molecular association.

Despite numerous efforts, there is currently no broadly effective vaccine developed to protect pigs against PRRSV [9]. Novel approaches need to be developed to control PRRS in pigs. The inhibition of key PPIs between PRRSV and CD163 with natural or synthetic compounds is a unique approach against the PRRS panzootic, that can be utilized as an adjuvant to vaccination to diminish further viral loads and shedding. So far, there is no known biological activity reported for compound B7. The significant dosage-dependent inhibition by B7 to PRRSV infection of PAMs makes this compound and its derivatives clear candidates for evaluating further their in vivo efficacy in inhibiting PRRSV infection. Our findings indicate that the inhibitory function of B7 and its analogues depends on the intact 3-(morpholinosulfonyl)anilino or the 3-(piperidinylsulfonyl)anilino moiety, which provides further clues for developing even more potent compounds against PRRSV infection. This finding, along with a full understanding of the key residues in CD163-SRCR5 domain involved in PRRSV recognition, would provide pertinent information for the targeted screen of effective compounds against PRRS.

## Conclusion

Porcine reproductive and respiratory syndrome (PRRS) is a panzootic that causes billion-dollar annual losses to the global pork industry. Currently there is no effective vaccine treatment for PRRS. Macrophage-specific surface receptor CD163 is required for the infection of pigs by PRRS viruses (PRRSV). Through virtual screening, we obtained 74 small molecules predicted to target the scavenger receptor cysteine-rich domain 5 (SRCR5) of CD163. Through a cell-based bimolecular fluorescence complementation (BiFC) assay, we identified one compound among these small molecules (designated here as B7) that significantly blocks the interaction between the PRRSV glycoproteins and the CD163-SRCR5 domain. We further confirmed that compound B7 inhibits various PRRSV strains’ infection of porcine alveolar macrophages (PAMs), the primary target of PRRSV. Further functional analysis revealed that the 3-(morpholinosulfonyl)aniline moiety of B7 or the 3-(piperidinylsulfonyl)aniline moiety in a B7 analogue is important for this inhibition. Our study identified a novel strategy to potentially prevent PRRSV infection in pigs using small molecules.

## Supplementary information

**Additional file 1 Figure S1.** Western Blotting of the BiFC SRCR5-VN, SRCR2-VN, and vector VN proteins. **Figure S2.** CD163 target site (with residues shown) for virtual screening. **Figure S3.** MTT assay of the B7 compound incubated with PAMs for 24 h. **Figure S4:** Direct titration of PRRSV incubated with DMSO or B7. **Figure S5.** MTT assay of the B7 and its analogue compounds incubated with PAMs for 24 h. **Figure S6.** PRRSV binding assay. **Table S1.** B7 and B7 Analogue Compounds Screened. **Table S2.** The Sequences of Primers Used In This Study.

## Data Availability

The datasets used and/or analyzed during the current study are available from the corresponding author on reasonable request.

## Abbreviations

BiFC: Bimolecular fluorescence complementation
PAMs: Porcine alveolar macrophages
PPI: Protein-protein interaction
PRRS: Porcine reproductive and respiratory syndrome
PRRSV: PRRS viruse
qRT-PCR: Quantitative reverse transcription polymerase-chain-reaction
SRCR5: Scavenger receptor cysteine-rich domain 5
WB: Western blotting
