# Supplementary material for: Small molecules block the interaction between porcine reproductive and respiratory syndrome virus and CD163 receptor and the infection of pig cells
Source: Virol J. 2020 Jul 30;17:116. doi: 10.1186/s12985-020-01361-7 (PMC7392821; doi:10.1186/s12985-020-01361-7)
Supplement: Supplementary file 1 — Additional file 1 Figure S1. Western Blotting of the BiFC SRCR5-VN, SRCR2-VN, and vector VN proteins. Figure S2. CD163 target site (with residues shown) for virtual screening. Figure S3. MTT assay of the B7 compound incubated with PAMs for 24 h. Figure S4: Direct titration of PRRSV incubated with DMSO or B7. Figure S5. MTT assay of the B7 and its analogue compounds incubated with PAMs for 24 h. Figure S6. PRRSV binding assay. Table S1. B7 and B7 Analogue Compounds Screened. Table S2. The Sequences of Primers Used In This Study. [file 12985_2020_1361_MOESM1_ESM.docx]

**Chemical Compounds Block the Interaction between Porcine Reproductive and Respiratory Syndrome Virus and CD163 Receptor and the Infection of Pig Cells**

Chang Huang^1^, Denzil Bernard^2^, Jiaqi Zhu^1^, Radha C. Dash^4^, Alexander Chu^1^, Alec Knupp^1^, Anna Hakey^1^, Kyle M. Hadden^4^, Antonio Garmendia^3,*^, Young Tang^1,*^

**Supplementary Figures and Tables**

**Figure S1:** Western Blotting of the BiFC SRCR5-VN, SRCR2-VN, and vector VN proteins.

**Figure S2:** CD163 target site (with residues shown) for virtual screening.


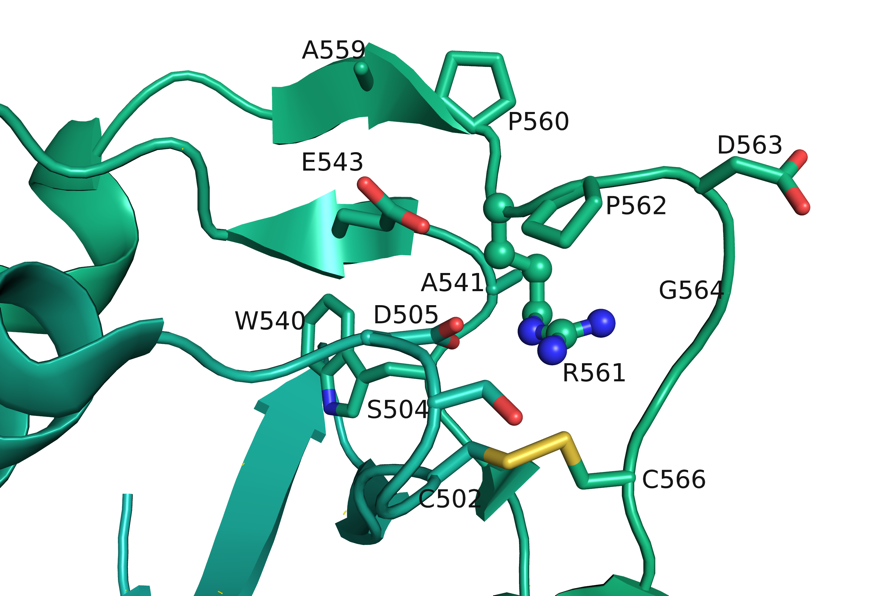


**Figure S3:** MTT assay of the B7 compound incubated with PAMs for 24 h. Scatter plots = mean±SD, n=3.

**Figure S4:** Direct titration of PRRSV incubated with DMSO or B7. PRRSV VR-2332 strain was incubated with DMSO as the Ctrl or 15 µM B7 at 37℃ for 1 h. The viruses were then serially diluted and directly subjected for titration assay. Bar = mean±SD, n=3.

**Figure S5:** MTT assay of the B7 and its analogue compound incubated with PAMs for 24 h. Bar = mean±SD, n=3.

**Figure S6:** PRRSV binding assay. PAMs were inoculated with VR2332 PRRSV at MOI of 0.1 for 1 h and treated concurrently with DMSO as the Ctrl or 15 µM B7. Subsequently, the cells were immediately washed and RNA extracted (0 hpi), or incubated without further treatment for 24 h before RNA extraction (24 hpi). qRT-PCR was performed to quantify the relative quantity of PRRSV RNA. Bar = mean±SD, n=3.

**TABLE S1: B7 and B7 Analogue Compounds Screened.**

| **Designated ID** | **Structure** | **Name** |
| --- | --- | --- |
| **B7** | **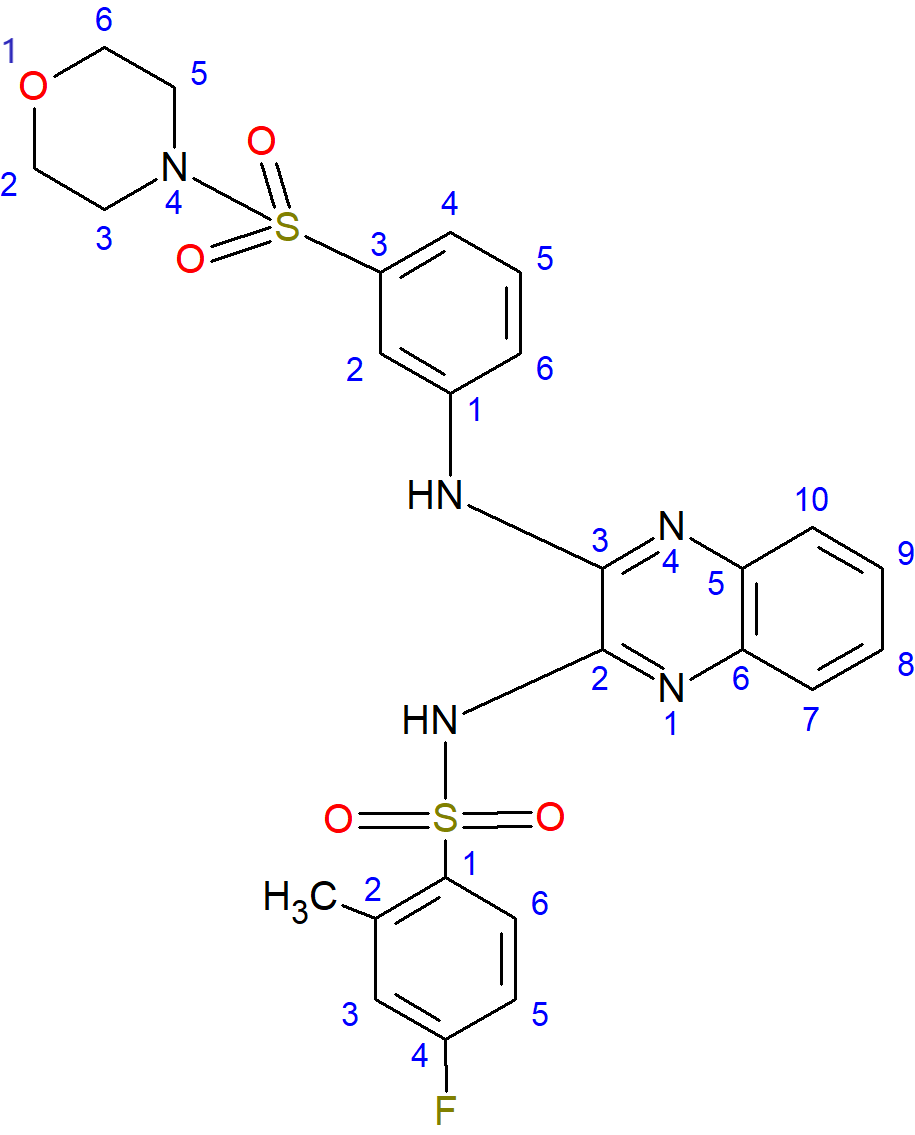** | **Molecular Formula: [C](https://pubchem.ncbi.nlm.nih.gov/)_[25](https://pubchem.ncbi.nlm.nih.gov/)_[H](https://pubchem.ncbi.nlm.nih.gov/)_[24](https://pubchem.ncbi.nlm.nih.gov/)_[FN](https://pubchem.ncbi.nlm.nih.gov/)_[5](https://pubchem.ncbi.nlm.nih.gov/)_[O](https://pubchem.ncbi.nlm.nih.gov/)_[5](https://pubchem.ncbi.nlm.nih.gov/)_[S](https://pubchem.ncbi.nlm.nih.gov/)_[2](https://pubchem.ncbi.nlm.nih.gov/)_**  **4-Fluoro-2-methyl-N-[3-(3-morpholin-4-ylsulfonylanilino) quinoxalin-2-yl] benzenesulfonamide** |
| **B7-A1** | **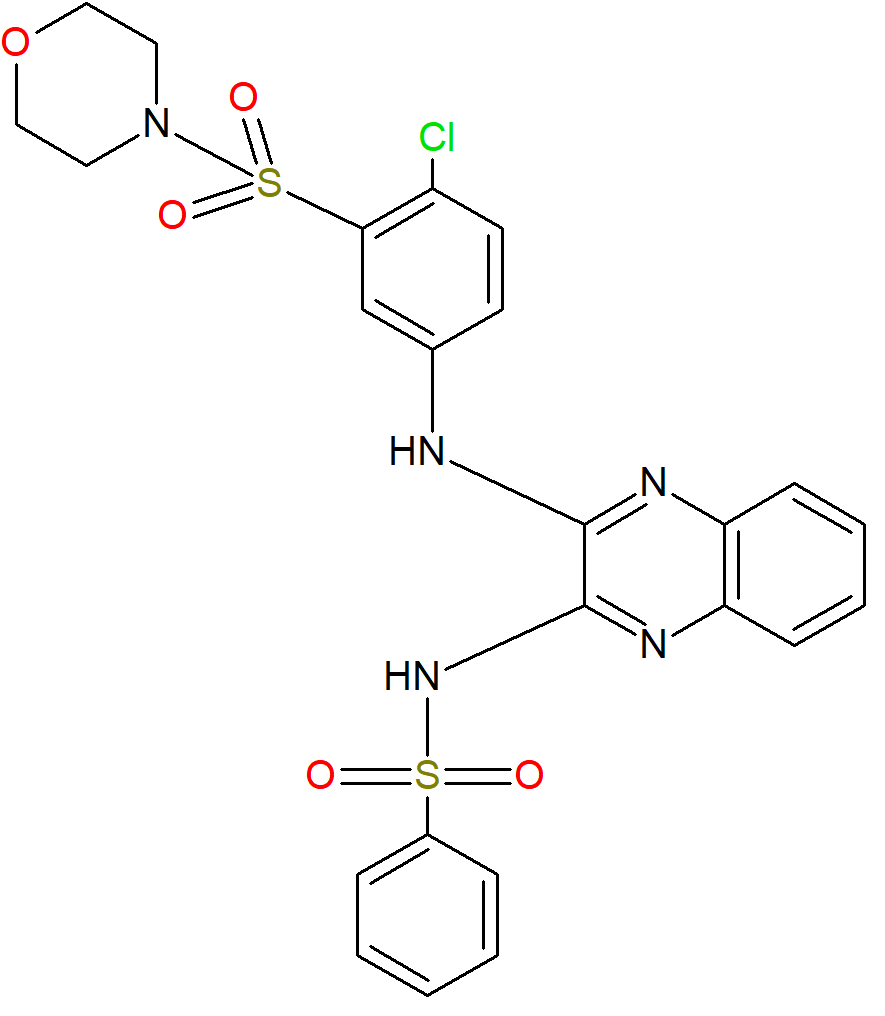** | **Molecular Formula: [C](https://pubchem.ncbi.nlm.nih.gov/)_[24](https://pubchem.ncbi.nlm.nih.gov/)_[H](https://pubchem.ncbi.nlm.nih.gov/)_[22](https://pubchem.ncbi.nlm.nih.gov/)_[ClN](https://pubchem.ncbi.nlm.nih.gov/)_[5](https://pubchem.ncbi.nlm.nih.gov/)_[O](https://pubchem.ncbi.nlm.nih.gov/)_[5](https://pubchem.ncbi.nlm.nih.gov/)_[S](https://pubchem.ncbi.nlm.nih.gov/)_[2](https://pubchem.ncbi.nlm.nih.gov/)_**  **N-[3-(4-Chloro-3-morpholin-4-ylsulfonylanilino)quinoxalin-2-yl] benzenesulfonamide** |
| **B7-A2** | **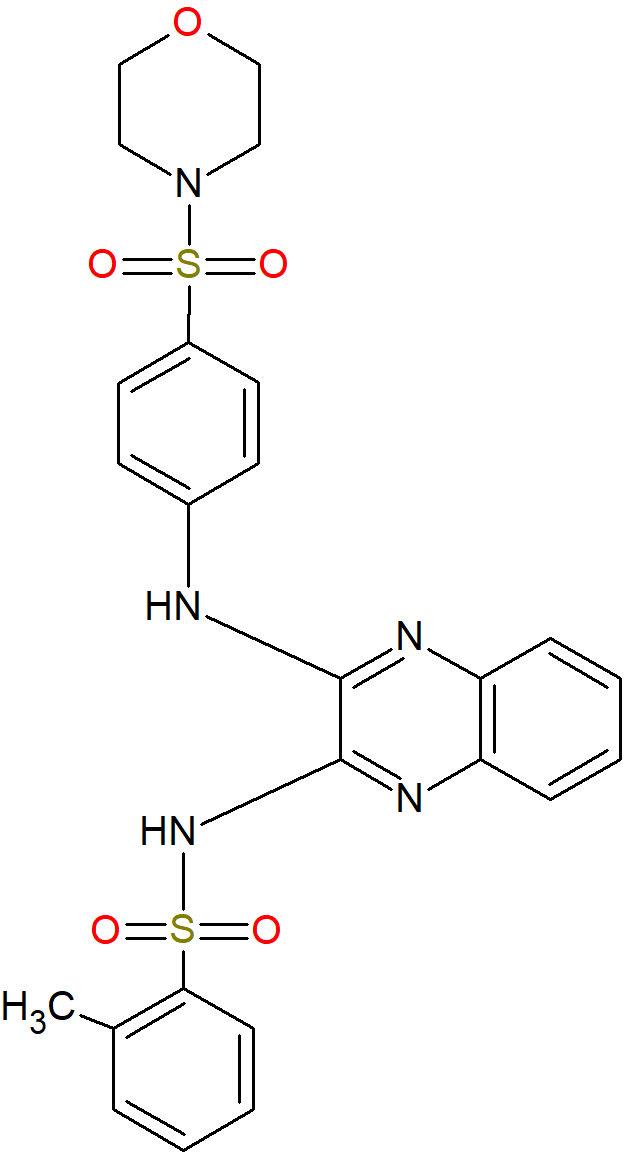** | **Molecular Formula: [C](https://pubchem.ncbi.nlm.nih.gov/)_[25](https://pubchem.ncbi.nlm.nih.gov/)_[H](https://pubchem.ncbi.nlm.nih.gov/)_[25](https://pubchem.ncbi.nlm.nih.gov/)_[N](https://pubchem.ncbi.nlm.nih.gov/)_[5](https://pubchem.ncbi.nlm.nih.gov/)_[O](https://pubchem.ncbi.nlm.nih.gov/)_[5](https://pubchem.ncbi.nlm.nih.gov/)_[S](https://pubchem.ncbi.nlm.nih.gov/)_[2](https://pubchem.ncbi.nlm.nih.gov/)_**  **2-Methyl-N-[3-(4-morpholin-4-ylsulfonylanilino)quinoxalin-2-yl] benzenesulfonamide** |
| **B7-A3** | **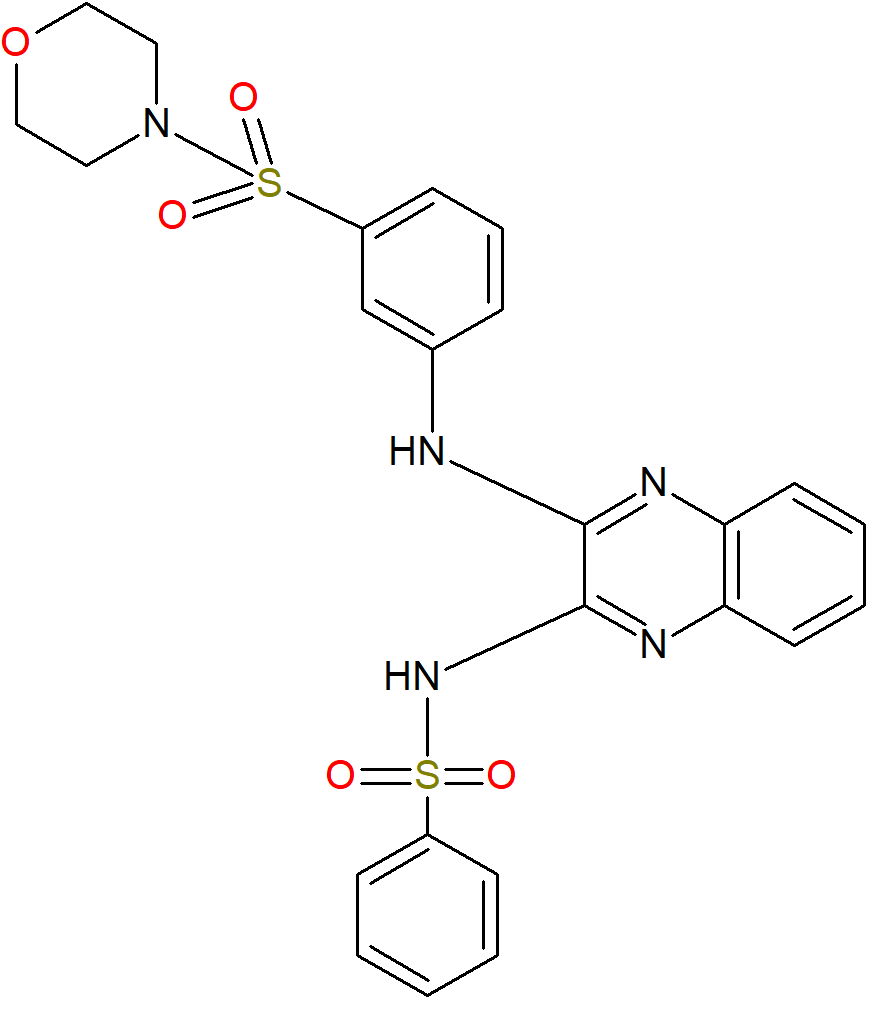** | **Molecular Formula:**  **[C](https://pubchem.ncbi.nlm.nih.gov/)_[24](https://pubchem.ncbi.nlm.nih.gov/)_[H](https://pubchem.ncbi.nlm.nih.gov/)_[23](https://pubchem.ncbi.nlm.nih.gov/)_[N](https://pubchem.ncbi.nlm.nih.gov/)_[5](https://pubchem.ncbi.nlm.nih.gov/)_[O](https://pubchem.ncbi.nlm.nih.gov/)_[5](https://pubchem.ncbi.nlm.nih.gov/)_[S](https://pubchem.ncbi.nlm.nih.gov/)_[2](https://pubchem.ncbi.nlm.nih.gov/)_**  **N-[3-(3-Morpholin-4-ylsulfonylanilino)quinoxalin-2-yl]**  **benzenesulfonamide** |
| **B7-A4** | **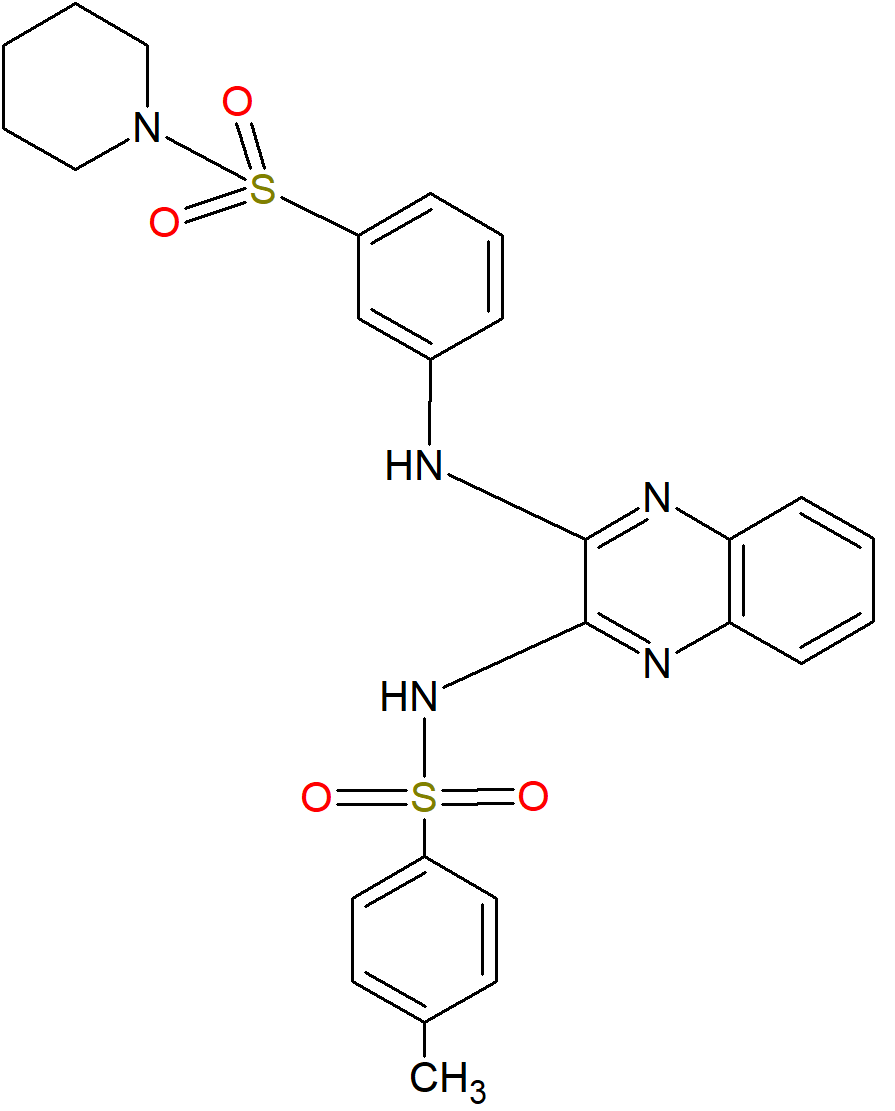** | **Molecular Formula:**  **[C](https://pubchem.ncbi.nlm.nih.gov/)_[26](https://pubchem.ncbi.nlm.nih.gov/)_[H](https://pubchem.ncbi.nlm.nih.gov/)_[27](https://pubchem.ncbi.nlm.nih.gov/)_[N](https://pubchem.ncbi.nlm.nih.gov/)_[5](https://pubchem.ncbi.nlm.nih.gov/)_[O](https://pubchem.ncbi.nlm.nih.gov/)_[4](https://pubchem.ncbi.nlm.nih.gov/)_[S](https://pubchem.ncbi.nlm.nih.gov/)_[2](https://pubchem.ncbi.nlm.nih.gov/)_**  **4-Methyl-N-[3-(3-piperidin-1-ylsulfonylanilino)quinoxalin-2-yl] benzenesulfonamide** |
| **B7-A5** | **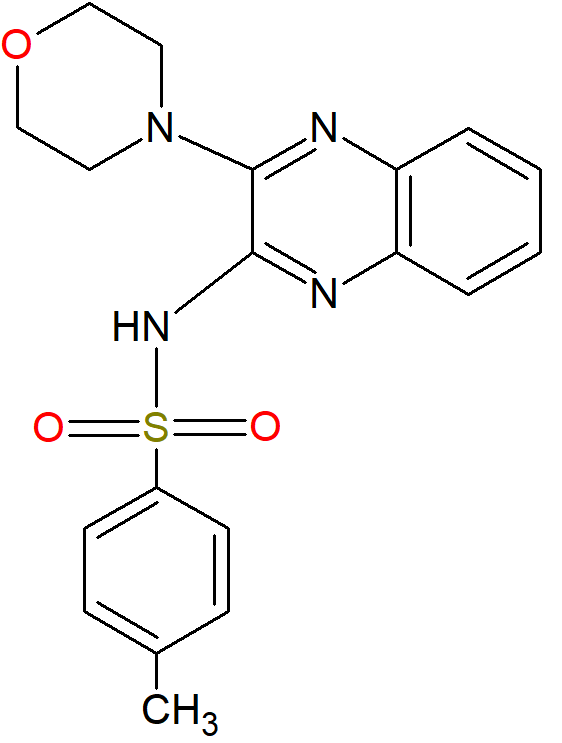** | **Molecular Formula:**  **[C](https://pubchem.ncbi.nlm.nih.gov/)_[19](https://pubchem.ncbi.nlm.nih.gov/)_[H](https://pubchem.ncbi.nlm.nih.gov/)_[20](https://pubchem.ncbi.nlm.nih.gov/)_[N](https://pubchem.ncbi.nlm.nih.gov/)_[4](https://pubchem.ncbi.nlm.nih.gov/)_[O](https://pubchem.ncbi.nlm.nih.gov/)_[3](https://pubchem.ncbi.nlm.nih.gov/)_[S](https://pubchem.ncbi.nlm.nih.gov/)**    **[(4-Methylphenyl)sulfonyl](3-morpholin-4-ylquinoxalin-2-yl) amine** |
| **B7-A6** | **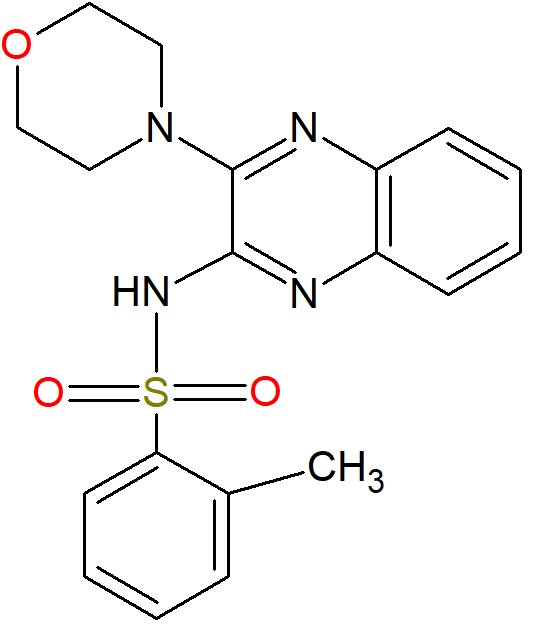** | **Molecular Formula:**  **[C](https://pubchem.ncbi.nlm.nih.gov/)_[19](https://pubchem.ncbi.nlm.nih.gov/)_[H](https://pubchem.ncbi.nlm.nih.gov/)_[20](https://pubchem.ncbi.nlm.nih.gov/)_[N](https://pubchem.ncbi.nlm.nih.gov/)_[4](https://pubchem.ncbi.nlm.nih.gov/)_[O](https://pubchem.ncbi.nlm.nih.gov/)_[3](https://pubchem.ncbi.nlm.nih.gov/)_[S](https://pubchem.ncbi.nlm.nih.gov/)**  **2-Methyl-N-(3-morpholin-4-ylquinoxalin-2-yl) benzenesulfonamide** |
| **B7-A7** | **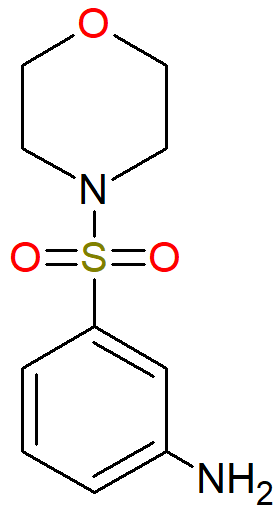** | **Molecular Formula:**  **C_10_H_14_N_2_O_3_S**  **3-(morpholinosulfonyl)anilino** |
| **B7-A8** | **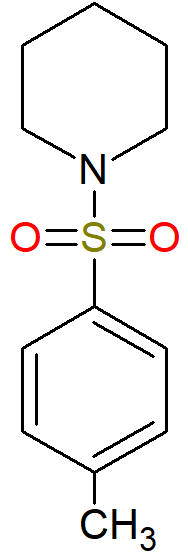** | **Molecular Formula:**  **C_12_H_17_NO_2_S**  **3-(piperidinylsulfonyl)anilino** |

**TABLE S2: The Sequences of Primers Used In This Study.**

| **Primers for qRT-PCR*^a^*** | **Sequence (5’ → 3’)** |
| --- | --- |
| **Lelystad-F** | **AAGATGACATCCGGCACCAC** |
| **Lelystad-R** | **CCGGCAGCATAAACTCAACCTG** |
| **VR2332-F** | **AAACCAGTCCAGAGGCAAGG** |
| **VR2332-R** | **GCAAACTAAACTCCACAGTGTAA** |
| **SDSU73-F** | **CCCTAGTGAGCGGCAATTGTGTC** |
| **SDSU73-R** | **GGCGCACAGTATGATGCGTC** |
| **NADC30-F** | **GGATGGCCAGCCAGTCAATC** |
| **NADC30-R** | **TGACGTCATCTTCAGTCGCTAGAG** |
| **GAPDH-F** | **CATCCTGGGCTACACTGAGG** |
| **GAPDH-R** | **GCTTGACGAAGTGGTCGTTG** |

***^a^*F, forward primer, R, reverse primer.**
